# Supplementary material for: AuthentiCT: a model of ancient DNA damage to estimate the proportion of present-day DNA contamination
Source: Genome Biol. 2020 Sep 15;21:246. doi: 10.1186/s13059-020-02123-y (PMC7490890; doi:10.1186/s13059-020-02123-y)
Supplement: Supplementary file 3 — Additional file 3. Review history. [file 13059_2020_2123_MOESM3_ESM.docx]

**Review history**

**First round of review**

**Reviewer 1**

The present-study is a very welcome modelling effort that describes new features of ancient DNA degradation with autocorrelation of the position of cytosine deamination in the center of sequences. This is used to improve on previous methods to estimate whether there a set of sequences comprise both an ancient (degraded) and contaminating (not degraded) molecules. This is a very worthwhile pursuit as it could be used to authenticate ancient genome data from any species, not just humans, and without large SNP reference data sets. A limitation at the present is that this method using single-stranded library-prepared data, which is still uncommon. The main questions for the study surround the rates of false positive and false negatives at the lower range of contamination (<10%), which in practice is very important to authenticate ancient genomes. The variance at this range in practice, for a range of human/non-human genomes, would benefit from being clarified.

The study seems to focus on estimating contamination accurately for substantially contaminated samples (e.g. the first tick on the x-axis in Figure 3 is 25% contamination-very high), whereas for the majority of the field of ancient DNA the goal is to be able to very confidently separate samples with e.g. <3% contamination from those with e.g. >5% contamination. However, Figure 3 looks quite worrying for this purpose, as e.g. Vindija 33.19 seems to get an overestimate by 3%, and Les Cottes an underestimate of 3%. Similar overestimates appear in Figure 5 for Goyet. To address this, it could be interesting if the authors could run their estimates on as many ssDNA ancient human data sets that they can find in the literature, some of which hominin males where X-chromosome contamination can be estimated for comparison (or Neandertals where ancestry is an approach that the authors use). One would want to see that the new method doesn't produce very variable results.

In Figure 6, particularly in the inset, it is clear that AuthentiCT and PMDtools perform really very similar for excluding contamination so as to only make a minor practical difference. In some ways this is surprising, as PMDtools just assume a simple fixed deamination rate without inferring it from the data. However, it seems that the method is geared to running on 10k or 100k reads, since the modelling is computationally extensive. Can it run on very large datasets such as full genomes with perhaps a billion of reads to exclude contamination akin to PMDtools?

**Reviewer 2**

Peyrégne and Peter present a new approach to estimate contamination rates in (human) ancient DNA data sets. Although a number of methods already exist, this is an important field of research and the proposed method not only uses a new aspect of ancient DNA deamination - non-independence of cytosine deamination events along a DNA-fragment - but also seems to perform quite favorable compared to other approaches. I have to point out, that I cannot judge the maths behind the approach, but despite this point, I found the manuscript well readible and easy to follow. The authors also acknowledge previous work in appropriate way, which unfortunately cannot be taken for granted in manuscripts these days.
I only have once major comment, but leave this as optional. The manuscript is very human-centered. While I indeed think that identifying contamination from related species is not really an issue for people working on mammoth, it is an issue for scientists working on domesticated species and there is a large number of these studies. Therefore, I think the relevance of the manuscript would increase substantially, if the authors would add examples using species beyond humans. While it is implicitly clear that the approach can be used on other species, I would really like to see this explicitly in the manuscript.

**Authors’ response to reviewers**

We thank both reviewers for their time and evaluation of our manuscript. We carried out the analyses they suggested and we give a point-by-point response to their comments below.

Reviewer #1: The present-study is a very welcome modelling effort that describes new features of ancient DNA degradation with autocorrelation of the position of cytosine deamination in the center of sequences. This is used to improve on previous methods to estimate whether there a set of sequences comprise both an ancient (degraded) and contaminating (not degraded) molecules. This is a very worthwhile pursuit as it could be used to authenticate ancient genome data from any species, not just humans, and without large SNP reference data sets. A limitation at the present is that this method using single-stranded library-prepared data, which is still uncommon. The main questions for the study surround the rates of false positive and false negatives at the lower range of contamination (<10%), which in practice is very important to authenticate ancient genomes. The variance at this range in practice, for a range of human/non-human genomes, would benefit from being clarified.

> Response: We thank the reviewer for their thoughtful evaluation of our manuscript and address these concerns the best we could in our answers below.

The study seems to focus on estimating contamination accurately for substantially contaminated samples (e.g. the first tick on the x-axis in Figure 3 is 25% contamination-very high), whereas for the majority of the field of ancient DNA the goal is to be able to very confidently separate samples with e.g. <3% contamination from those with e.g. >5% contamination.

> Response: Although the first tick on the x-axis is at 25%, we reported estimates from 5% to 95% in steps of 5% and AuthentiCT is applicable to data with low levels of contamination. We have added a more detailed description of the axis in the figure caption to make it clearer for the reader, by stating that “Added contamination (x-axis) ranges from 5% to 95% in steps of 5% (see Supplementary Figure S3 for a narrower range from 1 to 10% in steps of 1%).” We have also added Supplementary Figure S3 that provides a zoomed view of the estimates between 1% and 10%. Furthermore, we note that Figure 5 shows estimates for libraries with low levels of contamination.

However, Figure 3 looks quite worrying for this purpose, as e.g. Vindija 33.19 seems to get an overestimate by 3%, and Les Cottes an underestimate of 3%. Similar overestimates appear in Figure 5 for Goyet. To address this, it could be interesting if the authors could run their estimates on as many ssDNA ancient human data sets that they can find in the literature, some of which hominin males where X-chromosome contamination can be estimated for comparison (or Neandertals where ancestry is an approach that the authors use). One would want to see that the new method doesn't produce very variable results.

> Response: Using 19 single-stranded DNA libraries from ancient modern humans we compared the estimates from AuthentiCT to estimates obtained from X-chromosome sequences using the method from Moreno-Mayar et al. (2019). The results are very similar across contamination rates ranging from 0 to 12% (Figure 5, panel B), and consistent between different libraries from the same specimen; showing that AuthentiCT does not appear to produce very variable results. Note that Moreno-Mayar et al. (2019) report a tendency to slightly overestimate contamination for contamination levels below 10% (see section 3.4 in Moreno-Mayar et al., 2019) and we agree that estimating low levels of contamination accurately is a difficult problem. Overall, we compare contamination estimates for 50 libraries, and the 95% confidence intervals largely overlap in 48 cases (Figure 5A and 5B).

In Figure 6, particularly in the inset, it is clear that AuthentiCT and PMDtools perform really very similar for excluding contamination so as to only make a minor practical difference. In some ways this is surprising, as PMDtools just assume a simple fixed deamination rate without inferring it from the data.

> Response: We show in Figure 6B that the methods agree in classifying sequences with multiple C-to-T substitutions as ancient. A simple filter selecting sequences with 2 or more C-to-T substitutions would perform similarly for such sequences, as multiple C-to-T substitutions are exceedingly rare under the null model of sequencing errors and polymorphism. We now show the performance of filtering sequences solely based on the number of observed C-to-T substitutions in Supplementary Figure S5. It is not surprising to see the methods perform similarly for sequences with multiple C-to-T substitutions. The benefit of using PMDtools and AuthentiCT is their ability to better classify sequences with less than 2 C-to-T substitutions. In particular, AuthentiCT uses the proximity of non-deaminated Cs to C-to-T substitutions to gain further information about the most likely origin of that C-to-T substitution (Figure 6D) and fits parameter values to the data. Perhaps surprisingly, the biggest difference in likelihood is found in sequences with many non-deaminated Cs, which AuthentiCT is more confident to call as contaminant. This increases the ability to distinguish ancient and present-day DNA sequences (see Supplementary Figure S7), and is important to estimate contamination rates (which PMDtools is not designed to perform).

However, it seems that the method is geared to running on 10k or 100k reads, since the modelling is computationally extensive. Can it run on very large datasets such as full genomes with perhaps a billion of reads to exclude contamination akin to PMDtools?

> Response: AuthentiCT can be applied to full genomes. This is done by fitting the model parameters using a random subset of the sequences and then running the model on the larger dataset using a configuration file with the fitted parameter values. We showed that the fitting takes 3 to 10 minutes for 10,000 sequences (Supplementary Figure S9). We now added Supplementary Figure S10 showing how much time it took to run the fitted model on the large dataset from the Les Cottés Neandertal (average genomic coverage of 2.7-fold corresponding to ~100 million sequences produced by shotgun sequencing, Hajdinjak et al. (2018)). The running time is linear with the number of sequences, from ~2 minutes for 1 million sequences to 230 minutes for 100 million sequences. One could even make it faster by splitting the dataset by chromosomes and running it on multiple cores. We now mention this in the text (first paragraph in section 2.2.2).


Reviewer #2: ===
Peyrégne and Peter present a new approach to estimate contamination rates in (human) ancient DNA data sets. Although a number of methods already exist, this is an important field of research and the proposed method not only uses a new aspect of ancient DNA deamination - non-independence of cytosine deamination events along a DNA-fragment - but also seems to perform quite favorable compared to other approaches. I have to point out, that I cannot judge the maths behind the approach, but despite this point, I found the manuscript well readible and easy to follow. The authors also acknowledge previous work in appropriate way, which unfortunately cannot be taken for granted in manuscripts these days.

> Response: We thank the reviewer for this positive feedback on our manuscript.

I only have once major comment, but leave this as optional. The manuscript is very human-centered. While I indeed think that identifying contamination from related species is not really an issue for people working on mammoth, it is an issue for scientists working on domesticated species and there is a large number of these studies. Therefore, I think the relevance of the manuscript would increase substantially, if the authors would add examples using species beyond humans. While it is implicitly clear that the approach can be used on other species, I would really like to see this explicitly in the manuscript.

> Response: AuthentiCT can indeed be used for other species; for instance, our simulations relied on randomly generated sequences that are independent from the human genome. We now also carried a direct evaluation using real ancient sequences from bovids and equids (Supplementary Figure S4), and we do not detect contamination among the mapped sequences of these datasets. We also added a statement in the text (lines 89-90) that the method is applicable to other species.

**Second round of review**

**Reviewer 1**

I am satisfied with the authors' revision.

**Reviewer 2**

I have no further comments.
